# The involvement and possible mechanism of pro-inflammatory tumor necrosis factor alpha (TNF-α) in thoracic ossification of the ligamentum flavum

**DOI:** 10.1371/journal.pone.0178986

**Published:** 2017-06-02

**Authors:** Chi Zhang, Zhongqiang Chen, Xiangyu Meng, Mengtao Li, Li Zhang, Ann Huang

**Affiliations:** 1 Department of Orthopedics, Peking University International Hospital, Beijing, China; 2 Central Laboratory, Peking University International Hospital, Beijing, China; 3 Bone Research Laboratory, University of Texas Southwestern Medical Center, Dallas, Texas, United States of America; 4 Department of Orthopedics, Peking University Third Hospital, Haidian District, Beijing, China; 5 Department of Research, Daobio Inc., Dallas, Texas, United States of America; Augusta University, UNITED STATES

## Abstract

Thoracic ossification of the ligamentum flavum (TOLF) is characterized by ectopic bone formation in the ligamentum flavum and is considered to be a leading cause of thoracic spinal canal stenosis and myelopathy. However, the underlying etiology is not well understood. An iTRAQ proteomics was used to reveal the involvement of inflammation factors in TOLF. TNF-α is a pro-inflammatory cytokine implicated in the pathogenesis of many human diseases. Protein profiling analysis showed that the protein level of TNF-α increased in the ossified ligamentum flavum of TOLF, which was confirmed by western blot. The effects of TNF-α on primary ligamentum flavum cells was examined. Cell proliferation assay demonstrated that primary cells from the ossified ligamentum flavum of TOLF grew faster than the control. Flow cytometry assay indicated that the proportions of cells in S phase of cell cycle of primary cells increased after TNF-α stimulation. To address the effect of TNF-α on gene expression, primary cells were derived from ligamentum flavum of TOLF patients. Culture cells were stimulated by TNF-α. RNA was isolated and analyzed by quantitative RT-PCR. G1/S-specific proteins cyclin D1 and c-Myc were upregulated after TNF-α stimulation. On the other hand, osteoblast differentiation related genes such as Bmp2 and Osterix (Osx) were upregulated in the presence of TNF-α. TNF-α activated Osx expression in a dose-dependent manner. Interestingly, a specific mitogen-activated protein kinase ERK inhibitor U0126, but not JNK kinase inhibitor SP600125, abrogated TNF-α activation of Osx expression. This suggests that TNF-α activates Osx expression through the mitogen-activated protein kinase ERK pathway. Taken together, we provide the evidence to support that TNF-α involves in TOLF probably through regulating cell proliferation via cyclin D1 and c-Myc, and promoting osteoblast differentiation via Osx.

## Introduction

Ossification of ligamentum flavum (OLF) of the spine is characterized by ectopic bone formation in the ligamentum flavum, and it is more likely to affect Asian population, including the Chinese, Japanese and Korean [1–5]. It has been reported that OLF mostly occurs in the lower thoracic spine [4]. T10-11 and T11-12 have shown the highest occurrence rates. Thoracic ossification of the ligamentum flavum (TOLF) has been considered to be a leading cause of thoracic spinal stenosis and myelopathy [6]. One paper indicates that over 70% of patients suffering from thoracic spinal stenosis have been diagnosed with TOLF [7]. It has been suggested that some related factors are associated with TOLF, such as genetic factors, basic metabolic elements, and mechanical effects [8,9]. However, the underlying etiology of TOLF is not well understood.

Due to the differences in patient population of TOLF, some researches tend to define TOLF as a disease with a genetic susceptibility component. For example, among the Han Chinese population, two novel variants in the BMP-2 gene has been identified in TOLF patients [10]. In a case-control association study, four known single nucleotide polymorphisms (SNPs) of COL6A1 were genotyped among 338 Chinese Han subjects by high throughput GenomeLab SNPstream genotyping system [11]. It suggests that COL6A1 may be a common susceptibility gene for OLF in Chinese population. Despite the unknown mechanism of the SNP, another report also suggests one of bone-related genes Runx2 may be responsible for ectopic bone formation in the spinal ligament [12].

An association between levels of basic metabolic elements and TOLF has been studies [13,14]. The basic elements in the specimens have been examined in ossified ligamentum flavum [15]. This research group has demonstrated that the Ca content and the Ca/Mg ratio increase, while the Zn, Mn and Mo contents decrease in the ossification group. The Cu content and F content in the specimens of the ossification group are also higher. However, the mechanisms of the effects of basic metabolic elements on the ossification remain unknown. Some evidences have been provided to indicate the involvement of mechanical stress in the TOLF process [16,17]. A Epidemiology study has revealed a correlation between mechanical stress and the occurrence of TOLF [18]. One group studied thirty-four patients in whom decompressive surgery was performed for TOLF [19]. The data suggest that duration of preoperative symptoms may be the most important predictor of long-term surgery-related outcome in TOLF patients, while the type of ossified ligamentum flavum, dural adhesion, and simultaneous surgery for coexistent cervical or lumbar lesions seems not to affect the long-term postoperative prognosis. Mechanical stress has been reported to play a role in TOLF progression through the induction of osteogenic differentiation of TOLF cells [20,21]. Another research group showed that cyclic tensile strain applied to cultured cells from ossification of ligamentum flavum activated the ossification through the beta-catenin signaling pathway [22].

Effect of inflammatory processes on new bone formation has drawn more and more attention recent years. A tightly controlled inflammatory phase has been observed after fracture, which triggers repair cascade and is critical for bone remodeling [23,24]. Interestingly, transgenic animal model has indicated the important role of tumor necrosis factor-alpha (TNF-α) in fracture healing [25]. This suggests that TNF-α participates in promoting postnatal fracture repair and that processes of skeletal tissue development and postnatal repair are highly regulated by differing mechanisms. Heterotopic ossifications and several diseases with excessive bone formation have shown to be preceded by an inflammatory phase [26,27]. However, the effect of inflammation factors on TOLF is not clear.

In order to acquire a comprehensive and quantitative protein profile, the isobaric tags for relative and absolute quantitation (iTRAQ) has been developed to become an approach appropriate for investigating proteomic changes during various developmental stages [28].

The aim of the current study was to utilize iTRAQ-based quantitative proteomics to explore the causes of TOLF. We sought to assess the involvement and possible mechanism of the pro-inflammatory cytokine TNF-α in thoracic ossification of the ligamentum flavum.

## Materials and methods

### Patients and ligament samples

Patients were recruited in the Department of Orthopedics, Peking University Third Hospital. This study was approved by the Ethics Committee of Peking University Third Hospital, and complied with the Declaration of Helsinki (PUTH-REC-SOP-06-3.0-A27, # 2014003). Written informed consent was obtained from each participating patient. As for the TOLF patients, normal ligamentum flavum in adjacent levels from the same patient served as the self-control group for proteomic study. TOLF and control ligaments were aseptically collected from patients during the operation. The proteins of ossified and normal ligamentum flavum were extracted.

### Protein extraction and iTRAQ

After protein extraction, the protein concentration was determined by following the manufacture’s protocol with Braford method (Bio-Rad laboratories, Hercules, California, USA). Each sample 200 μg was added into 4 μl Recuding Reagent, then mixed with 2 μl Cysteine-Blocking Reagent. 100 μl Dissolution Buffer in iTRAQ kit was added. The solution in the bottom was discarded. After three times repetition of this step, a new collection tube was used to mix 4 μg trypsin with the acquired solution. The 50 μl Dissolution Buffer5 was combined with the above solutions, reaching 100 μl digested sample. The samples were labeled with iTRAQ reagents by adding the contents of the iTRAQ Reagent-8Plex Multiplex Kit, and the samples were then labeled with iTRAQ following the manufacture’s protocol (Applied Biosystem). As a result, proteins with iTRAQ ratios ≥ 1.5 were considered to be up-regulated, and proteins with iTRAQ ratios ≤ 0. 67 were considered to be down-regulated.

### Primary ligamentum flavum cell culture

The primary cells of ligamentum flavum were obtained by the tissue explant method as previously described [20]. Briefly, ligamentum flavum in the control normal site or the ossified site was aseptically dissected from TOLF patients during surgery, and the surrounding tissues were carefully removed under a dissecting microscope. After dissection, the ligaments were rinsed with PBS. The specimens were minced into approximately 0.5-mm^3^ pieces, washed twice with PBS, and then digested with 0.25% trypsin (Gibco, Grand Island, NY, USA) for 1 h at 37°C followed by 200 U/ml type I collagenase (Sigma-Aldrich, St. Louis, MO, USA) for 4 h at 37°C. The sections were cultured in DMEM (Hyclone, Logan, UT, USA) containing 10% FBS, 100 U/ml penicillin, and 100 μg/ml streptomycin (Gibco) at 37°C and 5% CO_2_. Explant-derived cells were passaged by 0.25% trypsin digestion. Passage 2 cells were used for the stimulation experiments. Photos of cells were taken by Olympus CKX41.

### Protein purification and Western blot

Protein was purified by acetone precipitation from the cell lysates as previously described [29]. Briefly, the protein pellet was dissolved in 1% SDS buffer, and centrifuged for 5min at 14000 rpm. Proteins were separated on 10% SDS-PAGE gels and transferred to a PVDF membrane followed by Western blot. 8% non-fat milk in TBS containing 0.1% Tween-20 was used to block non-specific binding. The blot was then incubated with an anti-TNF-α rabbit monoclonal antibody (1:200, Abcam, Cambridge, MA, USA), or an anti-β-actin rabbit monoclonal antibody (1:200, Abcam) at 4°C overnight followed by a secondary antibody (peroxidase-conjugated anti-rabbit IgG 1:5000, Millipore Corporation). An Azure C300 chemiluminescence system (Dublin, CA, USA) was used for detection.

### Cell proliferation assay

The primary ligamentum flavum cells from the control normal site or the ossified site of TOLF patients were seeded in 96-well culture plates at initial density of 1×10^4^ cells/well overnight. Cell proliferation was determined using the Cell Counting Kit-8 (CCK8) kit (Dojindo, kumamoto, Japan) according to the manufacturer’s instructions. At each time point, the cells were stained with 10μl CCK8 dye in 90μl culture medium for 2 h at 37°C. Then microplate reader (Tecan Infinite m200, Tecan, wetzlar, Germany) was used to measure the absorbance at 450 nm. All experiments were performed in triplicates.

### Flow cytometry

The cell cycle phases of cultured cells were determined using flow cytometry. A total of 10^5^–10^6^ ligamentum flavum cells were collected, washed three times with ice-cold PBS, and fixed with 70% ice-cold ethanol overnight. Before analysis, the cells were stained with 7-aminoactinomycin D (7-AAD) (20 μg/ml−1) (BD) in the presence of RNase (100 μg/ml−1). After no more than 30 min, the cells were collected and filtered by 300 meshes, followed by analysis on a FACSAria II flow cytometer (BD Special Order System). Modfit LT 3.2 software (BD Special Order System) was used to calculate the percentage of phases of cell cycle.

### Reverse transcription-quantitative PCR (RT-qPCR)

Primary ligamentum flavum cells was stimulated by TNF-α (100 ng/ml or as indicated; R&D Systems) for 24 hr before harvest. Total RNA was extracted from cultured cells using TRIzol reagent (Invitrogen Life Technologies, Carlsbad, CA, USA) as previously described [30]. The purity and integrity of the total RNA were verified using the RNA 6000 Nano assay with an Agilent Bioanalyzer 2100 (Agilent Technologies, Santa Clara, CA). One μg total RNA was reverse transcribed into cDNA using the GoScript Reverse Transcription System (Promega Corp., Madison, WI, USA). Specific primers for Bmp2, Osx, Cyclin D1 and c-Myc were designed using Primer Premier (Premier Biosoft, Palo Alto, CA, USA) and ordered by Sangon Biotech (Shanghai, China). qPCR was carried out in triplicate using SYBR-Green SuperReal PreMix Plus (Tiangen Biotech (Beijing) Co., Ltd., Beijing, China) and the iQ5 PCR system (Bio-Rad Laboratories, Inc., Hercules, CA, USA). The following reaction conditions were used: 95°C for 30 sec, and 40 cycles of 95°C for 5 sec and 60°C for 34 sec. Data were represented as cycle threshold (Ct) values. The RNA levels were compared using the 2-^ΔΔ^Ct method and were normalized to glyceraldehyde-3-phosphate dehydrogenase (GAPDH) levels.

### Statistical analysis

All qPCR experiments were repeated for at least three times. The data was reported as the mean ± standard deviation (S.D.). Comparisons were made between groups by Student’s t test, and p<0.05 was considered to be significant.

## Results

### The level of TNF-α protein increased in TOLF

**Fig 1:**
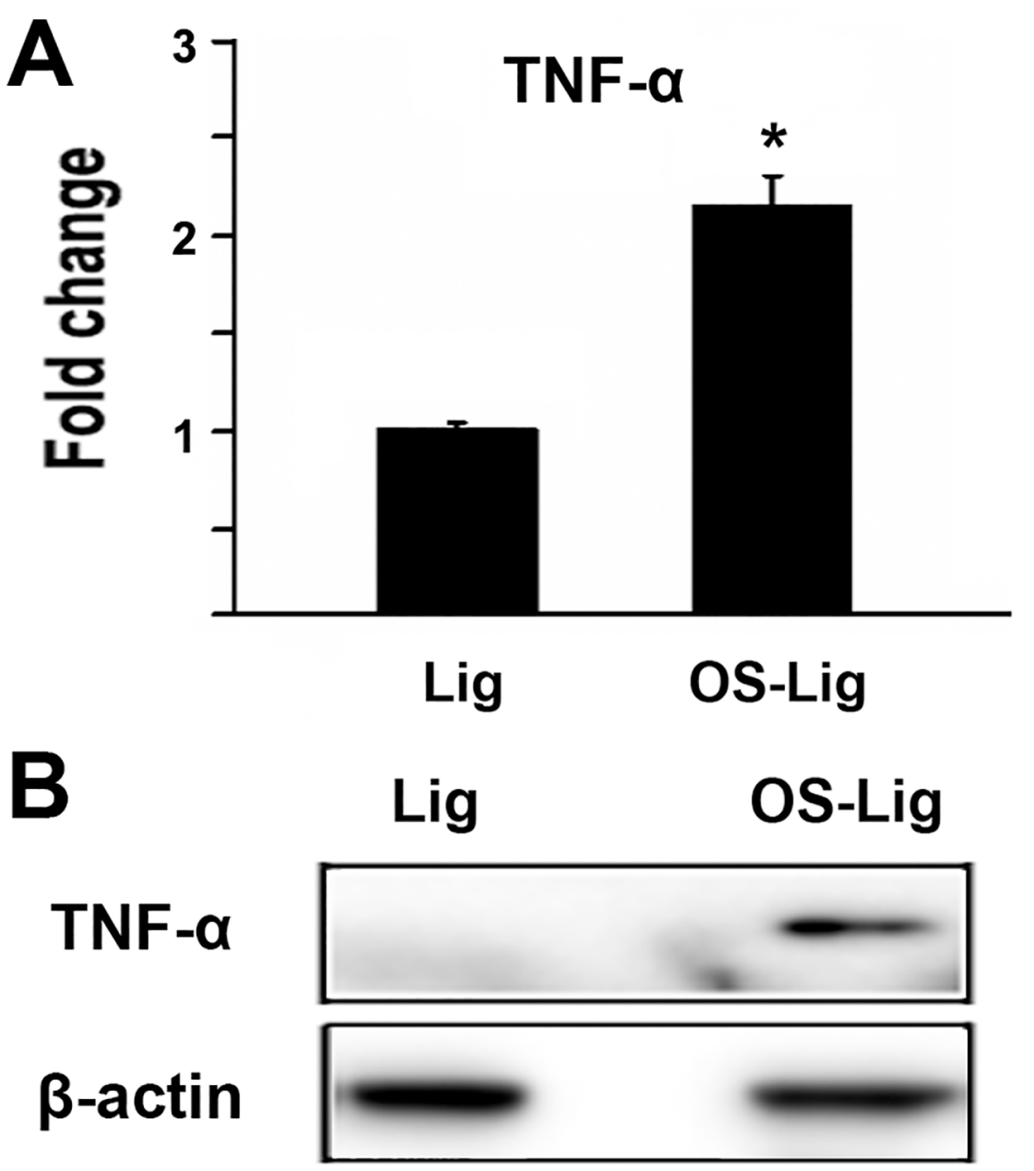
The level of TNF-α protein increased in TOLF. A) Fold change of TNF-α protein in the ossified ligamentum flavum of TOLF. Proteins were extracted from the ossified ligamentum flavum (OS-Lig) and normal ligamentum flavum (Lig). The samples were then labeled with iTRAQ following the manufacture’s protocol. *: A star indicates statistical significance compared to control group with p <0.05; B) TNF-α protein increased in primary cells of the ossified ligamentum flavum of TOLF by western blot. Primary cells were derived from the ossified ligamentum flavum (OS-Lig) and normal ligamentum flavum (Lig). Protein was purified and detected by western blot. An anti-TNF-α rabbit monoclonal antibody (1:200) or an anti-β-actin rabbit monoclonal antibody (1:200) was used.

iTRAQ-based quantitative proteomics was utilized to investigate protein profile of ligamentum flavum in patients with TOLF. These were evaluated by the fold change cutoff ratio ≥1.5 for up-regulation and ≤0.67 for down-regulation. There were totally 282 proteins identified to be differentially expressed. Among these proteins, ten inflammation-related factors were identified, including Tumor necrosis factor, Insulin-like growth factor II, Insulin-like growth factor-binding protein 5, Prostaglandin reductase 1, Latent-transforming growth factor beta-binding protein 3, Transforming growth factor beta-1, Neutrophil elastase, Serum amyloid A-4 protein, Protein S100-A9, and Prostaglandin-H2 D-isomerase. Several reasons led us to focus on the effect of TNF-α on TOLF in the current study. First of all, our preliminary data showed that the serum TNF-α of patients with TOLF was significantly higher compared with the control (data not shown); Secondly, our preliminary results of gene Microarray indicated that the gene expression level of TNF-α also increased in the ossified ligamentum flavum in TOLF (data not shown), supporting our proteomic observation in this study; Thirdly, previously reports have suggested an important role of TNF-α in fracture healing in transgenic animal model [25] or a promoting effect of TNF-α on osteogenic differentiation from mesenchymal stem cells [31]. As shown in Fig 1A, iTRAQ-based quantitative proteomics demonstrated that TNF-α protein expression was up-regulated by 2.1 fold in the ossified ligamentum flavum in TOLF patients. To confirm this observation, protein was purified from the cell lysates of primary cells of the ossified ligamentum flavum, and TNF-α protein expression was detected by western blot. As shown in Fig 1B, the level of TNF-α protein increased in the ossified ligamentum flavum compared with that in normal ligamentum flavum. β-actin was used as a negative control.

### Primary cells grew faster in the ossified ligamentum flavum of TOLF

**Fig 2:**
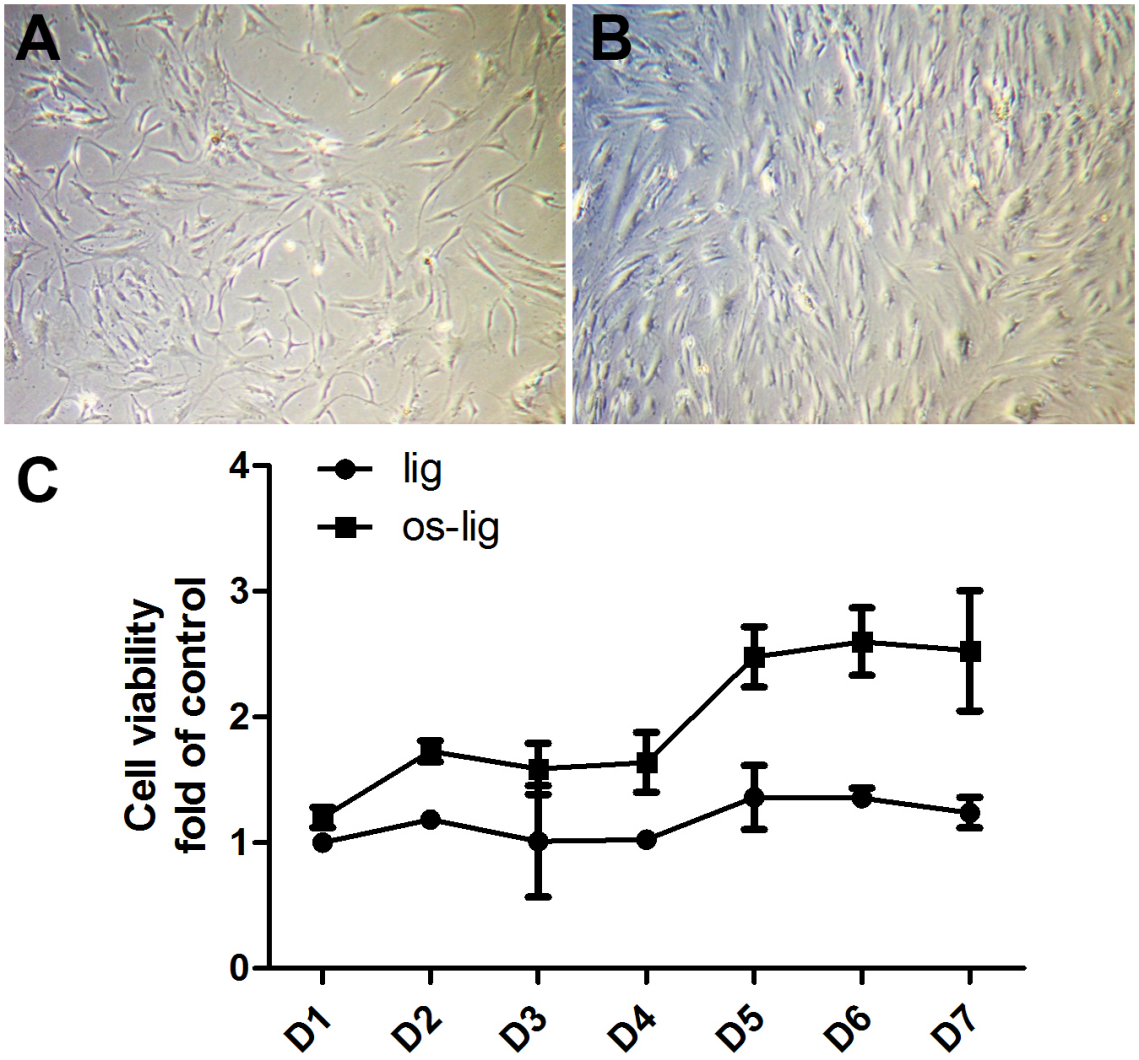
Primary cells grew faster in the ossified ligamentum flavum of TOLF. A) Cell morphology of primary cells from the normal ligamentum flavum in TOLF on Day 6; B) Cell morphology of primary cells from the ossified ligamentum flavum in TOLF on Day 6; C) Primary cells grew faster in the ossified ligamentum flavum in TOLF. The primary ligamentum flavum cells from normal site (Lig) or the ossified site (OS-Lig) of TOLF patients were seeded in 96-well culture plates at initial density of 1×10^4^ cells/well overnight, and cultured from Day 1 to Day 7. Cell proliferation was determined using the Cell Counting Kit-8 (CCK8) kit according to the manufacturer’s instructions.

To examine primary cell growth, the primary cells of ligamentum flavum from TOLF patients were obtained by the tissue explant method as described in Method Section. Cells were cultured for seven days, and cell growth of primary cells from the control normal site and the ossified site in TOLF were compared. The primary cells in two sites were long and spindle-shaped. Fig 2A showed a representative cell morphology from the control site on Day 6, and Fig 2B showed that from the ossified site in TOLF on Day 6. As shown in Fig 2C, primary cells were observed to grow faster in the ossified ligamentum flavum of TOLF compared with the control.

### Proportions of cells in S phase increased after TNF-α stimulation

**Fig 3:**
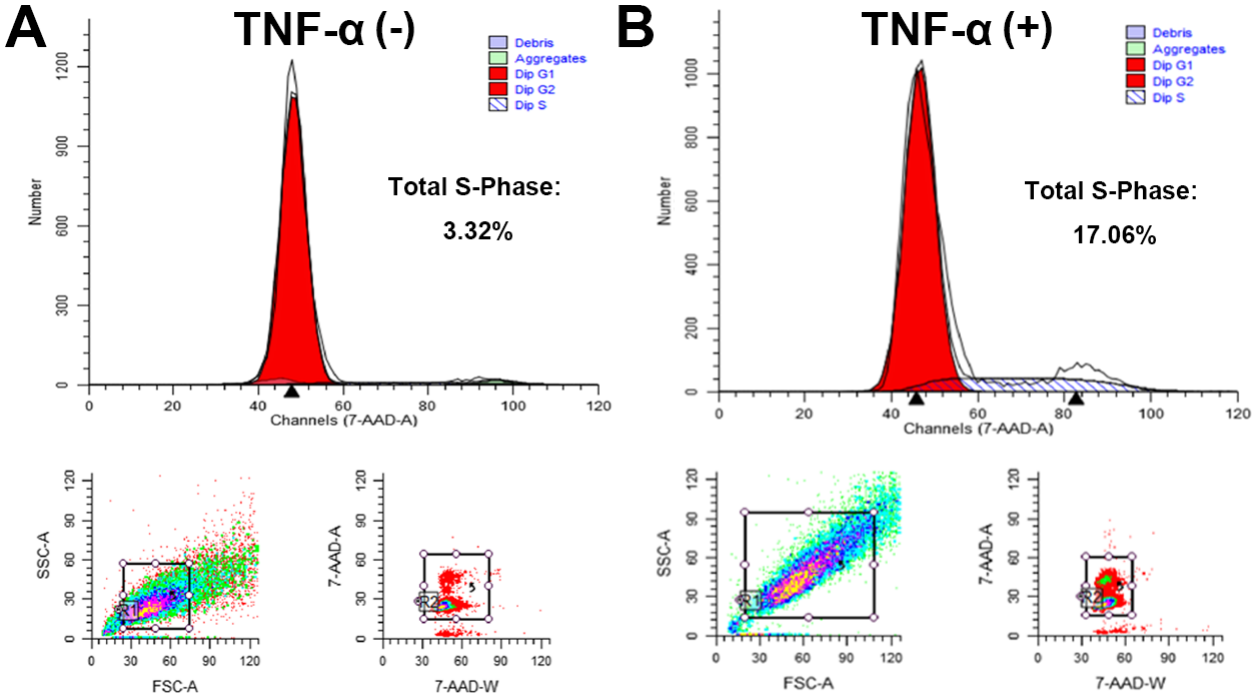
Proportions of cells in S phase increased after TNF-α stimulation. Primary cells of the ossified ligamentum flavum were cultured in the absence of TNF-α (A) or presence of TNF-α (B). Flow cytometry assay were used to examine the cell cycle. The proportions of cells in S phase of cell cycle increased up to 17.06% after TNF-α stimulation.

We found that the level of TNF-α protein increased in the ossified ligamentum flavum, and that primary cells of the ossified ligamentum flavum grew faster. Since the proliferation of cells is mainly regulated by the cell cycle, next we asked what was the effect of TNF-α on cell cycle, especially on S phase. Primary cells of the ossified ligamentum flavum were cultured in the absence or presence of TNF-α. Flow cytometry assay were used to examine the cell cycle. As indicated in Fig 3, compared with the control of 3.32% without TNF-α stimulation (Fig 3A), the proportions of cells in S phase of cell cycle of primary cells increased up to 17.06% after TNF-α stimulation (Fig 3B). This suggests that TNF-α may promote cell cycle progression via G1-S transition.

### TNF-α activated the expressions of the G1/S-specific protein cyclin D1 and c-Myc

**Fig 4:**
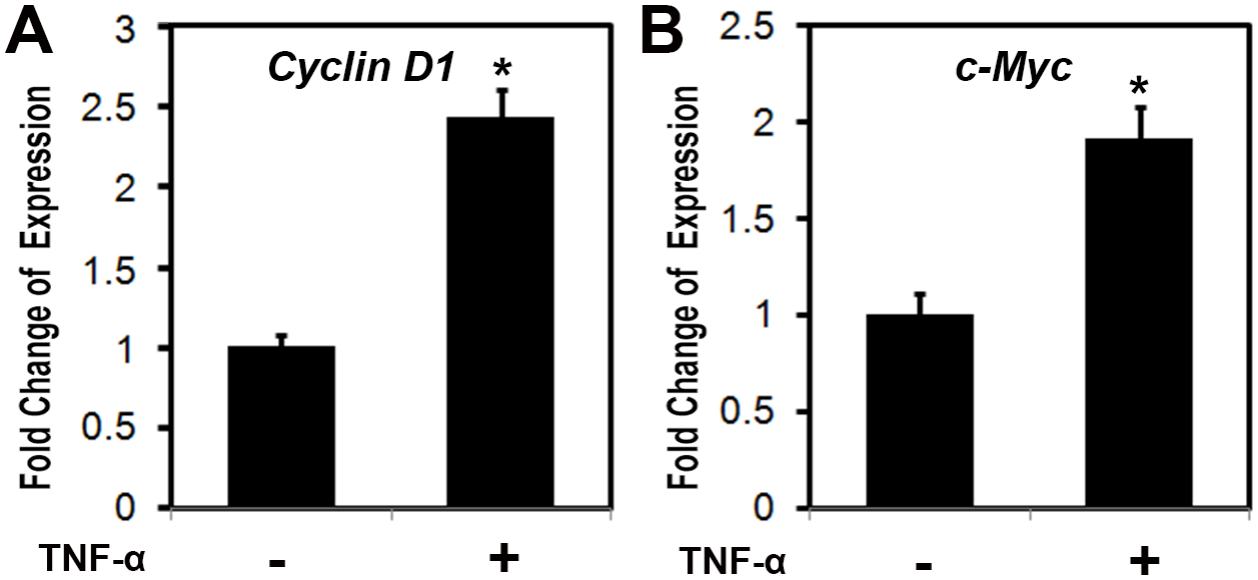
TNF-α activated the expressions of cyclin D1 and c-Myc. Primary ligamentum flavum cells derived from TOLF patients were cultured and treated with TNF-α (100 ng/ml) for 24 hr. Total RNA was isolated and measured by real time RT-PCR. The RNA level from the control group was normalized to a value of 1. Values were presented as the mean ±S.D. A paired *t*-test was performed comparing control and TNF-α group. *: A star indicates statistical significance compared to control group with p <0.05. A) Effect of TNF-α on cyclin D1 expression; B) Effect of TNF-α on c-Myc expression.

Next we determined the effect of TNF-α on G1/S-specific protein cyclin D1 and c-Myc. Primary ligamentum flavum cells derived from TOLF patients were cultured and treated as indicated with recombinant human TNF-α (100 ng/ml; R&D Systems) for 24 hr. Total RNA was isolated and measured by real time RT-PCR. As shown in Fig 4A, the expression of cyclin D1 increased by 2.45 fold after TNF-α stimulation. Fig 4B indicated that the expression of c-Myc was upregulated by 1.8 fold in the presence of TNF-α.

### TNF-α induced the expression of osteoblast genes in primary cells from TOLF

**Fig 5:**
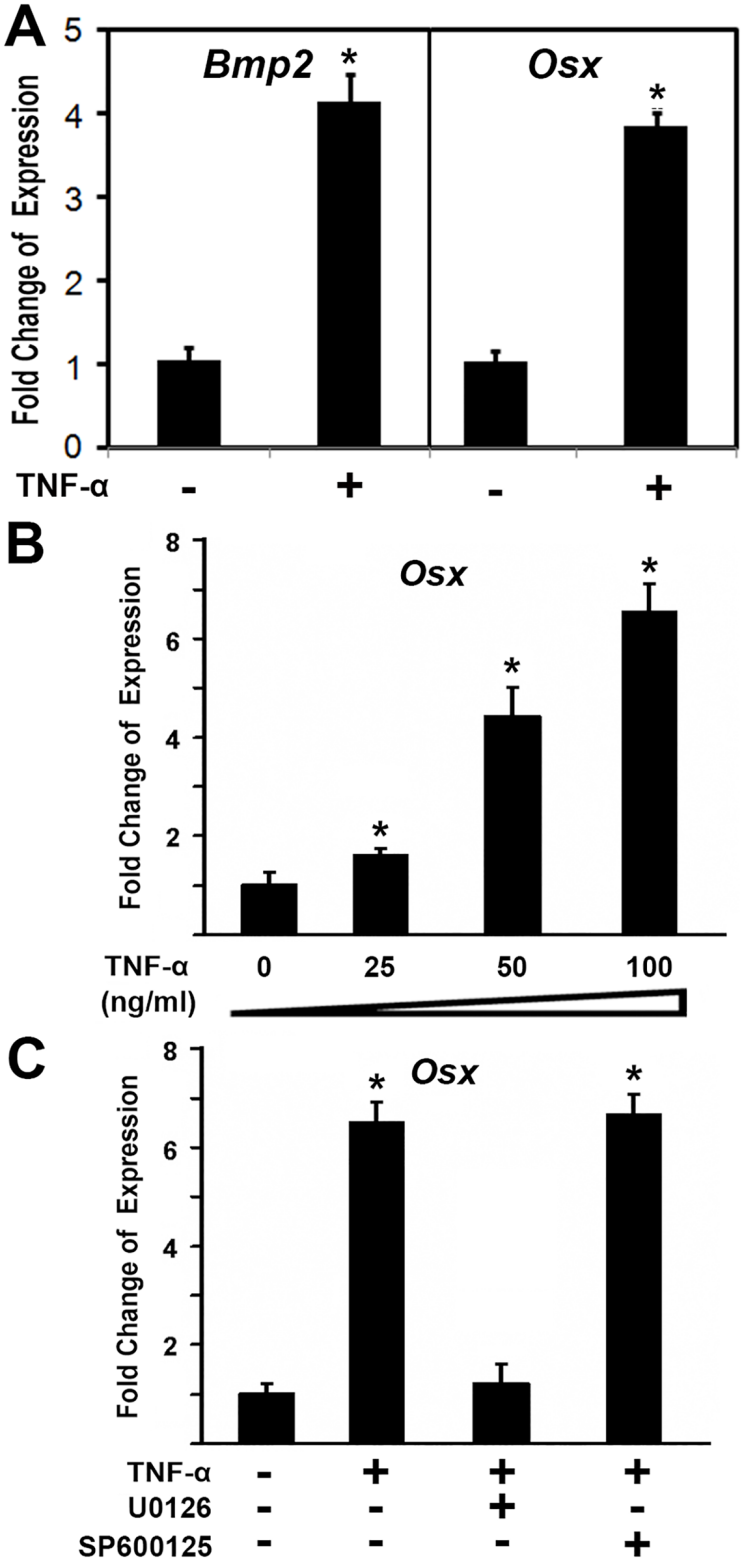
TNF-α induced the expression of osteoblast genes in primary cells from TOLF. Primary ligamentum flavum cells were derived from TOLF patients. Primary cells were treated with 100 ng/ml TNF-α or as indicated for 24 hr. Total RNA was isolated and measured by real time RT-PCR. The RNA level from the control group was normalized to a value of 1. Values were presented as the mean ±S.D. A paired *t*-test was performed comparing control and TNF-α group. *: A star indicates statistical significance compared to control group with p <0.05. A) Effect of TNF-α on the expressions of Bmp2 and Osx; B) TNF-α induced Osx gene expression in a dose-dependent manner; C) Fold change of the expression of Osx. Primary ligamentum flavum cells derived from TOLF patients were treated with 100 ng/ml of TNF-α for 24 h. U0126 and SP600125 were added to the culture medium 2 h before TNF-α treatment.

The level of TNF-α protein increased in TOLF (Fig 1), and it has been reported that TNF-α promotes osteogenic differentiation from mesenchymal stem cells [31]. These led us to hypothesize that TNF-α may participate in the ossification of ligamentum flavum in TOLF. To test our hypothesis, we examined the effect of TNF-α on ossification related gene expressions. Primary ligamentum flavum cells derived from TOLF patients were cultured and treated as indicated with recombinant human TNF-α (100 ng/ml; R&D Systems) for 24 hr. Total RNA was isolated and measured by real time RT-PCR. As shown in Fig 5A, the expression of bone morphogenetic protein 2 (Bmp2) increased by 4.1 fold after TNF-α stimulation, while osteoblast specific transcription factor Osterix (Osx) expression was upregulated by about 3.8 folds. In a separate experiment, primary cells were treated with increasing amounts of TNF-α. As shown in Fig 5B, the activation by Osx was detected when as low as 25ng/ml of TNF-α was added. Increasing amounts of TNF-α led to higher expression of Osx. Addition of 100ng/ml of TNF-α resulted in up to 6.48 fold activation of Osx. These observations demonstrated that TNF-α induced Osx gene expression in a dose-dependent manner in primary ligamentum flavum cells from TOLF patients.

Furthermore, we asked which pathway was involved in TNF-α induced Osx gene expression. To explore molecular mechanisms of TNF-α effect on Osx expression in primary ligamentum flavum cells, we used a loss-of-function approach to examine possible pathways involved. The following selective inhibitors were used: U0126 is a specific ERK inhibitor in mitogen-activated protein kinase pathway, and SP600125 is a specific inhibitor for JNK kinase pathway. Primary ligamentum flavum cells were treated with 100 ng/ml of TNF-α. Different inhibitors were added in the culture medium as indicated. As shown in Fig 5C, TNF-α treatment led to Osx expression increase by 6.5-fold. Addition of 50 μM U0126 almost abolished the increment of Osx expression induced by TNF-α. TNF-α-induced Osx activation remained unchanged by treatment with 50 μM SP600125 as shown in Fig 5C. These data suggest that TNF-α-induced Osx activation is mediated through the mitogen-activated protein kinase ERK pathway.

## Discussion

TOLF is characterized by pathological ectopic ossification in ligamentum flavum that generally consists of fibrous tissues. It has been considered to be a leading cause of thoracic spinal stenosis and can occur in multiple segments [32]. Although some studies have reported many related factors, including genetic factors, basic metabolic elements, and mechanical effects, for the time being the causes of TOLF are not well understood yet. As far as we know, this is the first study to discover the involvement and possible mechanism of TNF-α in thoracic ossification of the ligamentum flavum.

iTRAQ analysis uncovered 282 proteins which were identified to be differentially expressed in TOLF, including ten inflammation-related factors such as Tumor necrosis factor, Insulin-like growth factor II, Insulin-like growth factor-binding protein 5, Prostaglandin reductase 1, Latent-transforming growth factor beta-binding protein 3, Transforming growth factor beta-1, Neutrophil elastase, Serum amyloid A-4 protein, Protein S100-A9, and Prostaglandin-H2 D-isomerase. We focused on the effect of TNF-α on TOLF in this study.

TNF-α is a pro-inflammatory cytokine implicated in the pathogenesis of many human diseases. TNF-α interacts with its receptor to function through several pathways including the activation of nuclear factor kappa-B (NF-kB) involved in inflammation [33]. It is known that TNF-α has been associated with Ankylosing Spondylitis (AS) whose the unique hallmark is pathologic new bone formation [34]. It was reported that serum TNF-α level was significantly higher in AS patients compared with healthy controls [35]. The convincing evidence for a critical role of TNF-α comes from the transgenic mice study demonstrating that over-expression of TNF-α developed clinically spinal abnormalities similar to AS [36]. However, the underlying mechanisms still need to be elucidated. TOLF is characterized by ectopic bone formation in the ligamentum flavum. In this study, TNF-α was upregulated in both proteomic analysis and western blot experiments in primary cells from TOLF patients (Fig 1), which led us to further explore the effect of TNF-α on cell proliferation and osteoblast related genes.

Bone formation is a highly regulated developmental process, which involves mesenchymal stem cell differentiation into osteoblast lineage [37]. Osteoblast differentiation is controlled by different transcription factors such as Ihh, Runx2, and Osx [38]. Runx2 is essential for the mesenchymal stem cell differentiation into preosteoblasts [39]. Osx was discovered as a BMP2 induced gene in mouse pluripotent mesenchymal cells, encoding a transcription factor that is highly specific to osteoblasts, and *Osx*-null mice lacked bone completely [40]. Osx is required for osteoblast differentiation into mature osteoblast [40,41]. Both cell proliferation and differentiation are important for the process of bone formation. In this study, we demonstrated that TNF-α regulated cell proliferation via cyclin D1 and c-Myc. Primary cells from the ossified ligamentum flavum of TOLF grew faster than the control (Fig 2). The proportions of cells in S phase of cell cycle of primary cells increased after TNF-α stimulation (Fig 3). In addition, our results also showed that G1/S-specific proteins cyclin D1 and c-Myc were upregulated in the presence of TNF-α (Fig 4). It suggests that these data may partially explain the clinical observation of proliferous hypertrophy and hyperplasia of the ligamentum flavum in TOLF patient. On the other hand, our observations provide evidence that TNF-α activated the expression of osteoblast related genes. This is supported by the TNF-α stimulation experiments *in vitro*. TNF-α induced the expressions of Bmp2 and Osx in primary ligamentum flavum cells from TOLF (Fig 5A). TNF-α activated Osx expression in a dose-dependent manner (Fig 5B). More importantly, the regulation of Osx gene expression by TNF-α was abolished when the specific ERK inhibitor U0126 in mitogen-activated protein kinase pathway was added in primary cell cultures, thus indicating that TNF-α activates Osx gene expression through the mitogen-activated protein kinase ERK pathway. However, we cannot rule out other possible pathways involved in Osx regulation by TNF-α. Effect of TNF-α on osteoblast differentation has been controversial. Previous *in vitro* study showed that TNF-α inhibit osteoblast differentiation in MC3T3-E1-14 cells, and possible mechanism is by inhibiting the expression of insulin-like growth factor [42]. On the contrary, the data from other group demonstrated that TNF-α stimulation resulted in osteogenic differentiation of mesenchymal stem cells, and possible mechanism is by increasing TAZ expression [31]. One possible explanation could be that the dual effect of TNF-α on osteoblast differentiation might be dependent on the dose of TNF-α, specific cell type, different stages during cell differentiation, or the time of TNF-α presence.

In summary, iTRAQ-based proteomic approach were used to identify protein profile of ligamentum flavum in TOLF. The focus of this study revealed the involvement and possible mechanism of TNF-α in TOLF, which suggests the contribution of an inflammatory component to the cause of TOLF. We provided evidence to support that TNF-α involves in TOLF probably through regulating cell proliferation via cyclin D1 and c-Myc, and promoting osteoblast differentiation via Osx. There is a necessity of further studies to explore the molecular mechanisms of effects of different inflammation factors on ligamentum flavum in TOLF.

## Supporting information

S1 Table Primer sequences for qRT-PCR assays. (XLSX)

## Abbreviations

GAPDH: glyceraldehyde-3-phosphate dehydrogenase
iTRAQ: the isobaric tags for relative and absolute quantitation
Osx: Osterix
TNF: Tumor necrosis factor
TOLF: Thoracic ossification of the ligamentum flavum
